# Man’s best friend(s): Effects of a brief befriending meditation on human-animal relations

**DOI:** 10.1371/journal.pone.0278704

**Published:** 2022-12-16

**Authors:** Otto Simonsson, Simon B. Goldberg, Walter Osika

**Affiliations:** 1 Department of Clinical Neuroscience, Center for Psychiatry Research, Karolinska Institutet, Stockholm, Sweden; 2 Department of Sociology, University of Oxford, Oxford, United Kingdom; 3 Center for Healthy Minds, University of Wisconsin—Madison, Madison, Madison, WI, United States of America; 4 Department of Counseling Psychology, University of Wisconsin—Madison, Madison, WI, United States of America; 5 Department of Neurobiology, Center for Social Sustainability, Care Sciences and Society, Karolinska Institutet, Stockholm, Sweden; Beijing Normal University, CHINA

## Abstract

In two studies using samples representative of the US adult population with regard to age, sex and ethnicity, we investigated relationships between loving-kindness and compassion-based practices with speciesism, animal solidarity and desire to help animals. In a cross-sectional study (Study 1, N = 2,822), results showed that past 30 days practice and estimated lifetime number of hours of lovingkindness or compassion meditation were associated with more animal solidarity and greater desire to help animals. Past 30 days practice was also associated with less speciesism, but only when adjusting for sociodemographic characteristics. In an experimental study (Study 2, N = 1,102), results showed that participants randomized to a befriending meditation (a practice similar to loving-kindness and compassion meditation) condition scored higher on animal solidarity and desire to help animals than participants randomized to a control condition. No significant difference was observed on speciesism, but mediation analyses suggested that effects on all three outcomes were mediated through perceived commonality with animals.

## Introduction

The nature of the relationship between humans and animals has consequences not only for animal welfare. For instance, the treatment of animals has been shown to influence the incidence and spread of animal diseases [1], which can have downstream effects on human health (but see [2]). Other research has also shown that the consumption of meat and other animal products contributes to global heating and biodiversity loss [3,4]. It is therefore important, even if only for anthropocentric reasons, to better understand how more positive human-animal relations can be encouraged.

Previous research has investigated the effects of various psychological interventions on human-animal relations. For example, imagining positive contact with a specific individual animal has been found to increase perceived commonality with the animal’s subgroup and induce greater prosocial intentions toward animals in general [5] (see also [6]). The imagined intergroup contact approach has been shown to reduce intergroup bias generally [7,8], with some evidence to suggest that perceived commonality with the outgroup may be a key mediator [9,10]. However, it may be difficult to scale such interventions in real-world settings and have large numbers of people voluntarily choose to imagine positive interactions with animals. Other psychological interventions that might be easily accessible and more commonly used should therefore be investigated.

Loving-kindness and compassion-based practices such as befriending meditation [11] might be an alternative to imagined intergroup contact. Such practices are similar to imagined intergroup contact in that different types of people are typically brought to mind. However, imagined intergroup contact involves imagining positive interactions with outgroup members while loving-kindness and compassion-based practices typically involve directly cultivating a sense of kindness and friendliness toward whoever has been brought to mind [12,13]. The evidence to date suggests that these types of practices can reduce political intergroup bias [14] (see also [15]), with effects mediated by perceived commonality with the political outgroup [16]. Other research has also shown that such practices may increase social and nature connectedness [17–19], but the relationship between loving-kindness and compassion-based practices and human-animal relations remain largely unexplored.

In two studies using online samples representative of the US adult population with regard to age, sex and ethnicity, we investigated relationship between loving-kindness and compassion-based practices and three measures related to human-animal relations: speciesism, animal solidarity and desire to help animals. In Study 1 (N = 2,822), we conducted a cross-sectional study to assess the associations between lifetime and current engagement with loving-kindness or compassion meditation and speciesism, animal solidarity and desire to help animals. We hypothesized that lifetime and current engagement with loving-kindness or compassion meditation would be negatively associated with speciesism and positively associated with solidarity with animals and desire to help animals. In Study 2 (N = 1,102), we conducted a pre-registered randomized controlled trial to investigate the effects of a brief befriending meditation on speciesism, animal solidarity and desire to help animals. We hypothesized that participants randomized to listen to a brief befriending meditation (a practice similar to loving-kindness and compassion meditation) would score lower on speciesism and higher on solidarity with animals and desire to help animals than participants randomized to listen to an active control condition. We also hypothesized that the effect of befriending meditation on speciesism, animal solidarity and desire to help animals would be mediated by perceived commonality with animals.

## Study 1

### Materials and methods

In Study 1, the sample was comprised of US residents (18 years or older) who were recruited in October 2021 through Prolific Academic (https://app.prolific.co), which is a platform that allows researchers to upload study advertisements and recruit participants for research. The platform offers a representativeness function–which was used in this study–that uses proportionate stratification on three census-matched factors–age (18–27, 28–37, 38–47, 48–57, 58+), sex (male, female), and ethnicity (White, Mixed, Asian, Black, Other)–to reflect the US adult population’s demographic distribution.

### Design and procedure

All participants gave informed consent digitally (“Please click ‘Next’ to consent to participate in this study”) and responded to questions about sociodemographic characteristics (e.g., educational attainment, annual household income), speciesism [20] (α = .87 in Study 1), animal solidarity [21] (α = .96 in Study 1), and desire to help animals [22] (α = .77 in Study 1). Participants were also asked to report whether they had ever tried any type of meditation. Those who reported lifetime meditation use were asked to report whether they had ever tried loving-kindness or compassion meditation, including Metta, Compassion Cultivation Training, and Cognitively-Based Compassion Training. Participants who reported having been exposed to loving-kindness or compassion meditation were asked to report, on average, over the past 30 days how many days per week they engaged with loving-kindness or compassion meditation (1 = 0 days, 2 = 1 day, 3 = 2 days, 4 = 3 days, 5 = 4 days, 6 = 5 days, 7 = 6 days, 8 = 7 days) and estimated lifetime number of hours of loving-kindness or compassion meditation (1 = 0–10, 2 = 11–100, 3 = 101–500, 4 = 501–1000, 5 = 1001–5000, 6 = 5001+). Participants who reported no exposure to loving-kindness or compassion meditation were coded as 0 for both past 30 days practice and estimated lifetime number of hours. If the participant completed the study, they received $2.20. Study procedures were determined to be exempt from review by the Institutional Review Board at the University of Wisconsin-Madison. All procedures performed involving human participants were in accordance with the ethical standards of the 1964 Helsinki declaration and its later amendments or comparable ethical standards.

### Statistical analyses

We used three linear regression models to evaluate associations between meditation-related variables and human-animal relations (i.e., speciesism, animal solidarity and desire to help animals). Model 1 did not adjust for covariates; Model 2 controlled for sociodemographic characteristics: age in years (18–25, 26–34, 35–49, 50–64 or 65 or older), gender (male, female, transgender/non-binary), ethnoracial identity (non-Hispanic White, non-Hispanic African American, non-Hispanic Native American/Alaska Native, non-Hispanic Native Hawaiian/Pacific Islander, non-Hispanic Asian, non-Hispanic more than one race, Hispanic), sexual orientation (heterosexual, bisexual, gay/lesbian, other), educational attainment (some high school or less, high school graduate or equivalent, some college/community college degree, Bachelor’s degree or higher), annual household income (less than US$20,000, US$20,000–49,999, US$50,000–74,999, US$75,000 or more), marital status (married/living with a partner/in a long-term relationship, widowed, divorced/separated, not married/single); Model 3 controlled for the same covariates as Model 2, plus other variables that may be relevant to human-animal relations: diet (omnivore, pescatarian, vegetarian, vegan), pet ownership (yes, no), political affiliation (Republican, Democrat, Independent, other, none), and nature visits (every day, one to several times per week, one to several times per month, one to several times per year, never).

## Results

Table 1 presents results from the three regression models testing the associations between meditation-related variables and human-animal relations. As demonstrated in the table, both past 30 days practice and estimated lifetime number of hours were modestly associated with more animal solidarity and greater desire to help animals in all three models. Past 30 days practice was also mostly associated with less speciesism, but only when adjusting for sociodemographic characteristics.

**Table 1:** Loving-kindness or compassion meditation practice and human-animal relations.

	Speciesism		Animal Solidarity		Desire to Help Animals	
	*β*	*p*	*β*	*p*	*β*	*p*
Model 1						
Past 30 days practice	-.02	.312	.09	< .001	.06	.001
Lifetime practice	-.02	.389	.09	< .001	.06	.001
Model 2						
Past 30 days practice	-.04	.031	.09	< .001	.07	< .001
Lifetime practice	-.03	.094	.10	< .001	.06	.001
Model 3						
Past 30 days practice	-.02	.174	.07	< .001	.05	.006
Lifetime practice	-.01	.497	.07	< .001	.04	.030

β = standardized coefficients. Note: Past 30 days practice and lifetime practice were entered into the models separately (see S1 Table in S1 File for descriptive statistics on past 30 days practice and lifetime practice).

## Discussion

In Study 1, results showed that both past 30 days practice and estimated lifetime number of hours were associated with more animal solidarity and greater desire to help animals in all three models. Past 30 days practice was also associated with less speciesism, but only when adjusting for sociodemographic characteristics (Model 2).

There were limitations with the research design in Study 1 that should be considered when interpreting the findings. First, it is possible that the relationships between meditation-related variables and human-animal relations were confounded by variables that were not controlled for in the regression models (e.g., religious beliefs). The generally lower coefficients in Model 3 (as compared to Model 1 and 2) show that the included control variables had a noticeable impact on the results. Second, potential mechanisms linking loving-kindness or compassion meditation with human-animal relations were not investigated. Third, temporal sequence could not be established due to the cross-sectional research design, which means that the data cannot be used to infer causality. We aimed to address these limitations in Study 2. In this experimental study, we investigated the effects of a brief befriending meditation on speciesism, animal solidarity and desire to help animals, in a sample representative of the US adult population with regards to age, sex and ethnicity. We also examined perceived commonality with animals as a candidate mechanism.

## Study 2

### Materials and methods

In Study 2, the sample was comprised of US residents (18 years or older) who were recruited in July 2022 through Prolific Academic. The representativeness function that was used in Study 1 was also used in Study 2. The design plan, sampling plan, hypothesis and variables for this study were all pre-registered on the Open Science Framework: https://osf.io/rnpag/. Sample size was determined *a priori* using power analysis. We assumed a small effect size and found that a sample size of 1060 participants would achieve 80% power to detect an effect size of d = 0.20 with an alpha of .0166 (this alpha was chosen based on the Bonferroni correction to adjust for multiple comparisons) using an independent t-test. We therefore aimed to recruit approximately 1100 participants.

### Design and procedure

We utilized a posttest-only randomized experimental design to test whether participants randomized into a befriending condition would score lower on speciesism and higher on animal solidarity and desire to help animals than participants randomized into an active control group. After giving their consent to partake in the study, participants were randomized to listen to a 10-minute befriending meditation (befriending condition) or to listen to an expert talking about meditation (control condition). Participants in the befriending condition were first instructed to bring friendship and kindness to themselves by repeating the phrase: “May I be free from suffering, may I be happy and healthy, may I have ease of being.” The participants were then instructed to bring friendship and kindness to a loved one, a stranger, a difficult person, and all living beings. Participants in the control condition were educated about mindfulness meditation, the neuroscience of mindfulness, and the evidence to date on mindfulness-based programs. The audio for both conditions were derived from recordings by Professor Mark Williams.

After listening to the audio recording, participants were presented with an attention check depending on which audio recording they had been assigned to (see Supporting Information for more information). Participants who gave the wrong answer on the attention check (n = 3) were excluded from data analyses. All participants also completed a manipulation check to ensure that the befriending condition had the desired effects. As a manipulation check, we assessed whether the befriending meditation induced feelings of kindness and good-will toward others more than the active control on a scale from 1 (Not at all) to 5 (Very much; see Supporting Information for more information).

After the attention and manipulation checks, participants were assessed on perceived commonality with animals on a scale from 1 to 7 using a modified version of the Inclusion of the Other in the Self Scale [23] (higher scores mean more perceived commonality; see Supporting Information for more information). Using the same measures described in Study 1, the participants were also assessed on speciesism (α = .88 in Study 2), animal solidarity (α = .97 in Study 2) and desire to help animals (α = .83 in Study 2). Participants were then asked to respond to questions about age, gender, first language, educational attainment, frequency of meditation practice, and equipment used to listen to the audio clip (see Supporting Information for more information) through Qualtrics. If the respondent completed the study, they received $1.74. Study procedures were determined to be exempt from review by the Institutional Review Board at the University of Wisconsin-Madison.

### Statistical analyses

As specified in our preregistration, we performed an independent samples t-test to assess whether there were significant differences in human-animal relations (i.e., speciesism, animal solidarity and desire to help animals) scores across the two conditions. We also performed a pre-registered mediation analysis using Hayes’ (2017) [24] PROCESS macro (Model 4) with 5,000 bootstrapped estimates to assess whether perceived commonality with animals mediated the effects of the befriending meditation on speciesism, animal solidarity and desire to help animals.

## Results

Although the participants were randomly assigned to one of the two conditions, we first examined whether the two groups were balanced on characteristics that could potentially influence the results. Independent samples t-tests revealed that there were significant differences across conditions in age (t(1100) = - 2.84, p = .005), with participants in the befriending condition reporting higher mean age in years (M = 46.57, SD = 16.09) than participants in the active control condition (M = 43.84, SD = 15.80). Chi-square test revealed that there were no significant differences across conditions in gender (χ2 (5, N = 1102) = 0.80, p = 977), having English as first language (χ2 (1, N = 1102) = 0.02, p = .896), education level (χ2 (7, N = 1102) = 7.97, p = .336), frequency of meditation practice (χ2 (6, N = 1102) = 8.67, p = .193), or the equipment used to listen to the audio clip (χ2 (2, N = 1102) = 4.67, p = .097).

### Manipulation check

As a manipulation check, we assessed whether the befriending meditation induced feelings of kindness and good-will toward others more than the active control condition. An independent samples t-test revealed that there was a significant difference between conditions in feelings of kindness and good-will toward others (t(1100) = -10.25, p < .001, d = 0.62), with participants in the befriending condition reporting more kindness and good-will toward others (M = 3.72, SD = 1.00) than participants in the control condition (M = 3.05, SD = 1.15). This suggests that the manipulation had the desired effects.

### Perceived commonality with animals

As specified in our preregistration, we assessed whether participants in the befriending condition would score higher on perceived commonality with animals than participants in the active control condition. An independent samples t-test revealed that there was a very small but significant difference between conditions in perceived commonality with animals (t(1100) = -1.98, p = .048, d = 0.12), with participants in the befriending condition reporting more kindness and good-will toward others (M = 4.65, SD = 1.70) than participants in the control condition (M = 4.45, SD = 1.71).

### Human-animal relations

As specified in our preregistration, we assessed whether participants in the befriending condition would score lower on speciesism and higher on animal solidarity and desire to help animals than participants in the active control condition. An independent samples t-test revealed that there was no significant difference between conditions in speciesism (t(1100) = 1.89, p = .059, d = 0.11). An independent samples t-test revealed that there was a significant difference between conditions in animal solidarity (t(1100) = -2.81, p = .005, d = 0.17), with participants in the befriending condition reporting more animal solidarity (M = 5.13, SD = 1.49) than participants in the control condition (M = 4.88, SD = 1.54). An independent samples t-test revealed that there was a significant difference between conditions in desire to help animals (t(1100) = -2.77, p = .006, d = 0.17), with participants in the befriending condition reporting more desire to help animals (M = 5.78, SD = 1.07) than participants in the control condition (M = 5.60, SD = 1.17). None of the effects were moderated by demographics or other variables (i.e., age, gender, first language, educational attainment, meditation practice, equipment). The results remain largely unchanged when running linear regression models controlling for demographics and other variables. The effects on animal solidarity and desire to help animals both survived corrections for multiple comparisons.

### Mediation

As specified in our preregistration, we performed a mediation analysis using Hayes’ (2017) [24] PROCESS macro (Model 4) with 5,000 bootstrapped estimates to examine whether perceived commonality with animals mediated the effects of the meditation intervention (treatment vs. control) on speciesism, animal solidarity and desire to help animals. A significant indirect effect was detected linking the meditation intervention with all three outcomes mediated through perceived commonality with animals. This was apparent when examining effects on speciesism (indirect effect B = -0.091, 95% bootstrapped confidence interval [CI] = -0.18, -0.0011, representing 56.3% of the total effect), animal solidarity (indirect effect B = 0.14, 95% CI = 0.0016, 0.27, representing 53.9% of the total effect), and desire to help animals (indirect effect B = 0.086, 95% CI = 0.0010, 0.17, representing 45.7% of the total effect).

### Discussion

In Study 2, results showed that participants randomized to the befriending condition scored higher on animal solidarity and desire to help animals than participants randomized to the active control condition. No significant difference was observed across conditions on speciesism, but mediation analyses suggested that the effects on all three outcomes (i.e., speciesism, animal solidarity and desire to help animals) were mediated through perceived commonality with animals.

There were limitations with the research design in Study 2 that should be considered when interpreting the findings. First, this study only included posttest measures of human-animal relations. While such a research design can avoid potentially sensitizing participants to study hypotheses, it is possible that posttest differences in speciesism, animal solidarity and desire to help animals were due to pre-test differences. Second, the immediate effects of a brief befriending meditation were only assessed. It might therefore be valuable for future studies to investigate the short- and long-term effects of meditation interventions on human-animal relations. Third, participants in the befriending condition were instructed to bring friendship and kindness to all living being, which may have led to expectancy or demand effects.

## General discussion

The present research investigated the links between loving-kindness and compassion-based practices such as befriending meditation and human-animal relations. In Study 1, results showed that both past 30 days practice and estimated lifetime number of hours were associated with more animal solidarity and greater desire to help animals. Past 30 days practice was also associated with less speciesism, but only when adjusting for sociodemographic characteristics. In Study 2, results showed that participants randomized to the befriending condition scored higher on animal solidarity and desire to help animals than participants randomized to the active control condition. No significant difference was observed across conditions on speciesism, but mediation analyses suggested that the effects on all three outcomes were mediated through perceived commonality with animals. Taken together, these results suggests that meditation practices designed to cultivate a sense of kindness and friendliness toward oneself and others can influence human-animals relations, with effects mediated by perceived commonality with animals. This may be especially promising in light of the growing use of meditation [25–27].

The results in this research project correspond with previous meditation studies on intergroup relations [28] and perceived commonality with others [16]. Even though the effects of a brief befriending meditation on human-animal relations may be small, meditation interventions are highly scalable and might therefore have meaningful effects when delivered to large groups of people through, for example, meditation apps, which are the most popular mental health apps [29] and the most common means of learning how to meditate [30]. It is also worth noting that the befriending meditation was a very minimal intervention and effects may be larger with ongoing or more intensive practice. Future research should investigate the relative effects of short and long-term meditation practice and could utilize a naturalistic study design to better understand the potential effects of meditation practice in society.

The findings open the possibility of investigating the effects on human-animal relations of other interventions that dissolve the perceived difference between self and others. For example, classic psychedelics such as psilocybin have recently been examined as a potential treatment for certain psychiatric disorders [31]. Such substances reliably reduce the sense of self at higher doses [32] and have been shown to increase nature connectedness [33], but recent evidence suggests it might also influence human-animal relations [34]. Future research should therefore examine the potential effects on human-animal relations of classic psychedelics and other interventions with similar mechanisms of action.

The present research project utilized two different research designs to evaluate the links between loving-kindness and compassion-based practices such as befriending meditation and human-animal relations. There are, however, limitations to consider when interpreting the findings in both studies. First, even though the samples were representative of the US adult population with regards to age, sex and ethnicity, they might not have been representative on other unmeasured variables. Second, the dependent variables were self-report measures which are vulnerable to response biases. Future research should recruit samples that are representative on more variables and utilize more objective measures of human-animal relations (e.g., willingness to donate money to organizations related to animal rights, behaviors associated with human-animal relations).

## Conclusions

In sum, the findings suggest that loving-kindness and compassion-based practices such as befriending meditation can influence human-animal relations, with effects mediated by perceived commonality with animals. The results contribute to a small but growing literature on human-animal relations and indicate that different types of meditation may be a highly scalable means to change attitudes toward animals, which may lead to more sustainable ways of living.

## Supporting information

S1 File (DOCX)
