# Supplementary material for: Man’s best friend(s): Effects of a brief befriending meditation on human-animal relations
Source: PLoS One. 2022 Dec 16;17(12):e0278704. doi: 10.1371/journal.pone.0278704 (PMC9757557; doi:10.1371/journal.pone.0278704)
Supplement: S1 File — (DOCX) [file pone.0278704.s001.docx]

**Supporting Information**

Attention Check

*Befriending Condition*

During the audio recording, I was instructed to…

1. generate feelings of kindness toward myself and others.
2. memorize numbers and dates.
3. stretch my body.

*Control Condition*

During the audio recording, I learnt about…

1. mindfulness and meditation.
2. sports and gymnastics.
3. politics and law.

Manipulation Check

How much did you generate feelings of kindness and good-will toward others during the recording you listened to?

1. Not at all
2. Only a little
3. To some extent
4. Quite a bit
5. Very much

Perceived Commonality with Animals

Which picture best describes your relationship with animals?


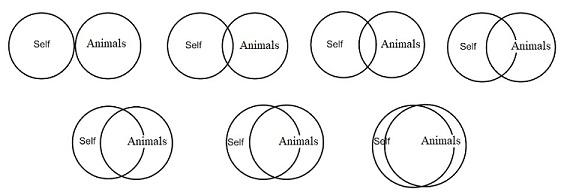


1. No overlap
2. Little overlap
3. Some overlap
4. Equal overlap
5. Strong overlap
6. Very strong overlap
7. Most overlap

Additional questions

What is your age?

- Slider scale

What is your gender?

- Male
- Female
- Transgender (male to female)
- Transgender (female to male)
- Non-binary gender
- Other (please specify)

Is English your first language?

- Yes
- No

What is the highest level of education you have completed?

- No formal qualifications
- Secondary education (e.g., GED/GCSE)
- High school diploma / A-levels
- Technical/community college
- Undergraduate degree (BA/BSc/other)
- Graduate degree (MA/MSc/MPhil/Other)
- Doctorate degree (PhD/other)
- Don’t know / not applicable

Please indicate how often you practice meditation, if at all.

- Never
- Once per month
- Two to three times per month
- Once per week
- Two to three times per week
- Four to six times per week
- Daily

What equipment did you use to listen to the audio clip played at the beginning of this session?

- Headphones
- Speakers
- Neither, I didn’t listen to the clip
- Other (please state)

| Table S1. Sample descriptive statistics | | | | | | |
| --- | --- | --- | --- | --- | --- | --- |
|  | Mean | SD | Min | Max | Skew | Kurtosis |
| Past 30 days practice | 0.51 | 1.44 | 0 | 8 | 3.3 | 13.6 |
| Lifetime practice | 0.39 | 1.04 | 0 | 6 | 3.0 | 12.3 |
| Note: 15% of the total sample reported lifetime use of lovingkindness or compassion meditation. | | | | | | |
